# A newborn with Cornelia de Lange syndrome: a case report

**DOI:** 10.1186/1757-1626-1-329

**Published:** 2008-11-19

**Authors:** Hakan Uzun, Dursun Ali Senses, Munevver Uluba, Kenan Kocabay

**Affiliations:** 1 Department of Pediatrics, Duzce University School of Medicine, Duzce, Turkey

## Abstract

Cornelia de Lange syndrome (CdLS) is a rarely seen multisystem developmental disorder syndrome characterized by facial dysmorphia (arched eyebrows, synophrys, depressed nasal bridge, long philtrum, down-turned angles of the mouth), upper-extremity malformations, hirsutism, cardiac defects, growth and cognitive retardation, and gastrointestinal abnormalities. The features of this disorder vary widely among affected individuals and range from relatively mild to severe. Early in life, the distinctive craniofacial features in mild de Lange syndrome may be indistinguishable from the severe (classical) phenotype. We present here a case of newborn with CdLs.

## Introduction

Cornelia de Lange syndrome (CdLS), also called Brachmann-de Lange syndrome, is a multiple congenital anomaly syndrome characterized by a distinctive facial appearance, prenatal and postnatal growth deficiency, psychomotor delay, behavioral problems, and malformations of the upper extremities. Cardiac defects and gastrointestinal anomalies are common, and many additional physical features occur, including myopia, palatal abnormalities, genitourinary abnormalities, congenital diaphragmatic hernias and hearing loss. Facial dysmorphism includes arched eyebrows, synophrys, short nose with anteverted nares, long philtrum, thin upper lip, and micrognathia [1,2]. The majority of the cases are sporadic, but a few cases showing an autosomal-dominant inheritance have been reported [3]. Although the exact incidence is unknown, CdLS likely affects 1 in 10,000 newborns [4]. This syndrome should be considered in the differential diagnosis of congenital anomalies and mental retardation, typical features of the presented a newborn with CdLS is discussed and the literature is reviewed.

## Case presentation

**Figure 1:**
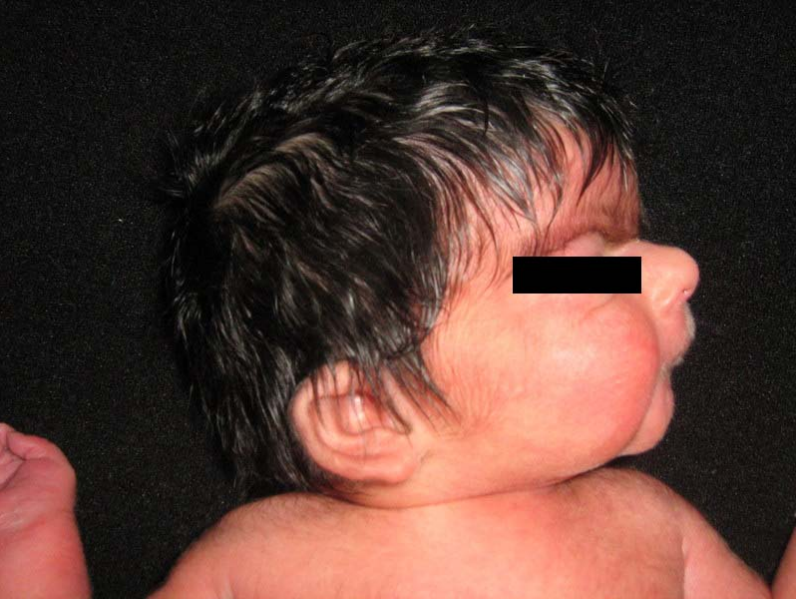
Appearance of facial features of case.

**Figure 2:**
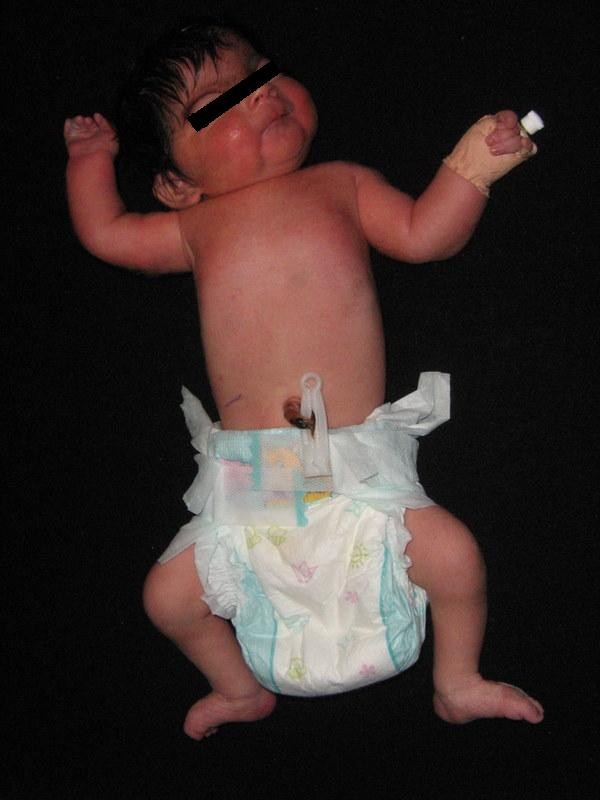
Appearance of the case.

A one-day old female newborn was referred to our hospital with the complaints of seizure and multiple congenital anomalies. She was the only child of a non-consanguineous marriage, born of a 37 weeks gestation normal vaginal delivery. On physical examination he had arched like confluent eyebrows and well-defined, long curly eyelashes, low anterior and posterior hairline, short neck, depressed nasal bridge, down-turned angles of the mouth and thin lips, cleft palate, microcephaly, excessive body hair (figure 1, 2), and small broad hands with simian creases, clinodactyly of left fifth fingers, short leg, hypertonicity, and small labia majora. On cardiac auscultation was heard 1–2/6 pansystolic murmur. Ophtalmologic examinations revealed normal findings.

Laboratory analysis including complete blood count, biochemical parameters and urinalysis were normal. Transthoracic echocardiography showed patent foramen ovale and patent ductus arteriosus. Cranial magnetic resonance imaging was normal. Chromosomal analysis was done on peripheral blood lymphocytes according to conventional techniques. The analysis revealed a normal female karyotype (46, XX).

## Discussion

The features of this disorder vary widely among affected individuals and range from relatively mild to severe. Based on the clinical variability in CdLS, Van Allen et al. [5] proposed a classification system. Type I, or classic, CdLS patients have the characteristic facial and skeletal changes of the diagnostic criteria established by Preus and Rex [6]. They have prenatal growth deficiency, moderate-to-profound psychomotor retardation, and major malformations, which result in severe disability or death. Type II, or mild, CdLS patients have similar facial and minor skeletal abnormalities to those seen in type I; however, these changes may develop with time or may be partially expressed. They have mild-to-borderline psychomotor retardation, less severe pre- and postnatal growth deficiency, and the absence of (or less severe) major malformations. Type III, or phenocopy, CdLS includes patients who have phenotypic manifestations of CdLS that are causally related to chromosomal aneuploidies or teratogenic exposures. Allanson et al. [2] in 1997 showed that, in the mild phenotype, the characteristic facial appearance may not appear until 2 to 3 years of age, while it is always present at birth in the classic phenotype. They also noted that the characteristic facial appearance decreased with time in the mild phenotype. In the same study the authors concluded that objective assessments supported the clinical impression of two distinct phenotypes, and those alternative discriminators, such as birth weight greater than 2,500 grams and absence of major limb anomalies, should be used to distinguish the mild from the severe phenotype early in life because of the similarity of facial features [2].

Mutations in the NIPBL, SMC1L1, and SMC3 genes cause CdLS. In 2004, two independent groups [7,8] found that 26–56% of patients with CdLS carry a heterozygous mutation of the NIPBL gene localized on 5p13.2. The NIPBL gene is the human orthologue of Drosophila Nipped-B and yeast Scc-2 and belongs to the family of chromosomal adherins involved in chromatid cohesion processes and enhancer-promoter communications [9,10]. The exact function of the human NIPBL gene product, called delangin, is unknown, but its wide expression pattern, including expression in embryonic limb bud, branchial arch, and craniofacial mesenchyme, is consistent with many of the anomalies observed in CdLS. An X-linked form of CdLS was reported in three male members from the same family and in one sporadic case, demonstrating the common combination of symptoms in the spectrum of CdLS, caused by mutations in the SMC1L1 gene which encodes a subunit of the cohesion complex [11]. The SMC1L1 gene provides instructions for making a protein that helps regulate the structure and organization of chromosomes. Recently, SMC3 encoding the other SMC cohesin component was found mutated in one patient with CdLS [12]. This gene provides instructions for making a protein that interacts with the SMC1L1 protein to regulate chromosome structure. In addition, a large number of reports have been described chromosomal abnormalities associated with CdLS, involving most chromosomes except for chromosomes 6, 15, 16, 19, 20 and 22 [13].

Genotype-phenotype correlations in the study of Gillis et al. [14] and Yan et al. [15] showed significant differences between patients with and without mutations in terms of the degree of growth retardation and developmental delay. In a different study on 39 sporadic cases of CdLS from the Netherlands, truncating NIPBL mutations were prevalently detected in CdLS patients of the classical type [16]. Musio et al., Deardorff et al. noted that both SMC3 and SMC1L1 mutation positive patients exhibit very mild facial dysmorphism, no absence or reduction of limbs or digits, and no other major structural anomalies [11,12].

The clinical phenotype of our patient is concordant with the classical type CdLS (distinctive facial appearance, prenatal growth retardation; expressed microcephaly and small hands) (Table 1). Analyses for mutations in the NIPBL, SMC1L1 and SMC3 genes are not currently available in Turkey. Therefore, genotype-phenotype correlation could not been performed our patient.

**Table 1:** Comparison of clinical features of CdLS in the present case

**Clinical features frequency in CdLS**	**Present case**
Prenatal onset growth retardation (68%)	+

Initial hypertonicity (100%)	+

Low-pitched weak cry in infancy (74%)	+

Feeding difficulties in the newborn period and infancy (71%)	+

Microbrachycephaly (93%)	+

Bushy eyebrows and synophrys (98%)	+

Long, curly eyelashes (99%)	+

Depressed nasal bridge (83%)	+

Anteverted nares (85%)	-

Down-turned angles of the mouth (94%)	+

High arched palate (86%) and reports of cleft palate	+

Micrognathia (84%)	-

Spurs in the anterior angle of the mandible, prominent symphysis (66%)	-

Short neck (66%)	+

Hirsutism (78%)	+

Low anterior and posterior hairline (92%)	+

Hypoplastic nipples and umbilicus (50%)	-

Micromelia (93%)	-

Phocomelia and oligodactyly (27%)	-

Clinodactyly of fifth fingers (74%)	+

Simian crease (51%)	+

Proximal implantation of thumbs (72%)	-

Hypoplastic external genitalia (57%),	+

Ophthalmologic manifestations (50%)	-

Cutis marmorata and perioral pale cyanosis (56%)	-

Seizures (23%)	+

Congenital Heart Defect (33%)	+

## Conclusion

We present one case of CdLS of neonatal diagnosis that we consider of interest due to the importance of an early recognition of the clinical condition for the family advice and the medical aid and for an appropriate development. Cornelia de Lange syndrome is a rare but well characterized syndrome. The key diagnostic features are the distinctive facial features, limb anomalies and growth retardation.

## Consent

Written informed consent was obtained from the patient for publication of this case report and accompanying images. A copy of the written consent is available for review by the Editor-in-Chief of this journal.

## Competing interests

The authors declare that they have no competing interests.

## Authors' contributions

HU, DAS, KK contributed to writing and preparation of manuscript. MU conveived the case report. All authors read and approved the final manuscript.
